# Garcinol Enhances TRAIL-Induced Apoptotic Cell Death through Up-Regulation of DR5 and Down-Regulation of c-FLIP Expression

**DOI:** 10.3390/molecules23071614

**Published:** 2018-07-02

**Authors:** Seok Kim, Seung Un Seo, Kyoung-Jin Min, Seon Min Woo, Ju-Ock Nam, Peter Kubatka, Shin Kim, Jong-Wook Park, Taeg Kyu Kwon

**Affiliations:** 1 Department of Immunology and School of Medicine, Keimyung University, 2800 Dalgubeoldaero, Dalseo-Gu, Daegu 42601, Korea; kimseok216@naver.com (S.K.); ssu3885@gmail.com (S.U.S.); Kyoungjin.min@gmail.com (K.-J.M.); woosm724@gmail.com (S.M.W.); god98005@dsmc.or.kr (S.K.); j303nih@dsmc.or.kr (J.-W.P); 2 Department of Food Science and Biotechnology, Kyungpook National University, Daegu 41566, Korea; namjo73@gmail.com; 3 Department of Medical Biology, Jessenius Faculty of Medicine, Comenius University in Bratislava, Martin 03601, Slovakia; kubatkap@gmail.com; 4 Department of Experimental Carcinogenesis, Division of Oncology, Biomedical Center Martin, Jessenius Faculty of Medicine, Comenius University in Bratislava, Martin 03601, Slovakia

**Keywords:** garcinol, TRAIL, c-FLIP, DR5, apoptosis

## Abstract

Garcinol is a polyisoprenylated benzophenone derived from the *Garcinia indica* fruit that possess potential therapeutic effects such as inhibition of inflammation and tumor expansion. Here, we investigated whether garcinol induces TRAIL sensitization in renal carcinoma cells. Single treatment with garcinol or TRAIL did not effect on apoptosis. However, combined treatment with garcinol plus TRAIL significantly induced apoptosis in renal carcinoma (Caki, ACHN and A498), lung carcinoma (A549), and hepatoma (SK-Hep1) cells. In contrast, garcinol plus TRAIL did not alter cell viability in normal cells. Garcinol plus TRAIL induced up-regulation of DR5 and down-regulation of c-FLIP expression at post-translational levels. Furthermore, knock-down of DR5 by siRNA and ectopic expression of c-FLIP blocked apoptotic cell death induced by garcinol plus TRAIL. Overall, our study provides evidence that garcinol can be exploited as a potential TRAIL sensitizer.

## 1. Introduction

Garcinol is a polyisoprenylated benzophenone which may be isolated from the dried rind of the fruit *Garcinia indica.* It exhibits multiple biological effects, such as anti-inflammatory, anti-microbial, and anti-oxidative activities [1,2,3]. Garcinol induces apoptosis via interference of the multiple signaling pathways, such as inactivation of STAT-3, NF-κB, and PI3K/Akt signaling pathways [4,5,6]. Garcinol also inhibits proliferation of tumor cells, angiogenesis, and cell cycle progression, and induces apoptosis via inhibition of NF-κB and cyclooxygenase-2 expression in oral cancer [7]. Furthermore, garcinol has a sensitizing effect in combined treatment with cisplatin or TRAIL [8,9]. However, the molecular mechanisms of this anti-cancer effect by garcinol are not well understood.

TRAIL selectively induces cell death in cancer cells [10,11]. However, a lot of cancer cells reveal resistance to TRAIL through multiple mechanisms, including down-regulation of death receptor (DR)4/5, and up-regulation of decoy death receptors and anti-apoptotic proteins [12,13,14]. New approaches to overcome TRAIL resistance that combined treatment with pharmacological agents that modify the function of tumor-dysregulated apoptotic genes have been investigated [15,16,17].

In this present study, we investigated the molecular mechanisms involved in the sensitizing effect of garcinol on TRAIL-induced apoptosis in cancer cells.

## 2. Results

### 2.1. Effect of Garcinol on TRAIL Sensitization

**Figure 1:**
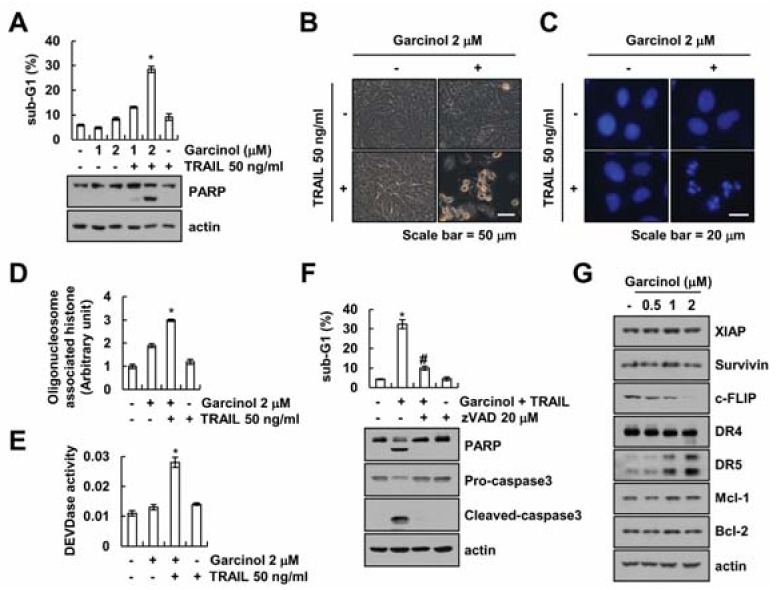
Garcinol sensitizes Caki cells to TRAIL-mediated apoptosis. (**A**–**E**) Caki cells were treated with garcinol (1–2 μM) and/or 50 ng/mL TRAIL for 24 h. Levels of apoptosis were assessed by flow cytometry, and western blot showing the PARP and actin (**A**). Morphology of cells was visualized with an optical microscope (**B**). DAPI staining detected condensation and fragmentation of nuclei (**C**). Detection of DNA fragmentation (**D**) and caspase activity (**E**). (**F**) Caki cells were treated with 2 μM garcinol and 50 ng/mL TRAIL for 24 h in the presence or absence of 20 μM z-VAD. Levels of apoptosis were assessed by flow cytometry, and western blot showing the PARP, pro-caspase-3, cleaved caspase-3 and actin. (**G**) Caki cells were treated with (0.5–2 μM) galcinol for 24 h. The related levels of proteins were detected by western blot using indicated antibody. * *p* < 0.01 compared to the control. # *p* < 0.01 compared to the garcinol plus TRAIL.

To investigate whether garcinol enhances TRAIL sensitization, we employed renal carcinoma Caki cells that are resistant to TRAIL. Cells were treated with garcinol alone (1 and 2 μM), TRAIL alone (50 ng/mL), and co-treatment with garcinol and TRAIL. We assayed amount of apoptotic cell death by sub-G1 population and PARP cleavage. Garcinol plus TRAIL increased the sub-G1 population and PARP cleavage (Figure 1A). However, single treatment with garcinol or TRAIL did not induce apoptosis. Garcinol plus TRAIL induced apoptotic morphologies, such as cell shrinkage, apoptotic body formation, and cell detachment on the plate (Figure 1B), nuclear condensation (Figure 1C), and the DNA fragmentation (Figure 1D). Furthermore, combined treatment with garcinol plus TRAIL increased caspase-3 activity (Figure 1E). To investigate the role of caspase activation in the garcinol plus TRAIL-induced apoptosis, we used a pan-caspase inhibitor, z-VAD-fmk. As shown in Figure 1F, z-VAD-fmk inhibited combined treatment-induced sub-G1 population and cleavage of PARP and caspase-3. Next, to investigate the molecular mechanism underlying the Caki cell death via combined treatment with garcinol and TRAIL, we analyzed the expression levels of apoptosis related proteins. Garcinol markedly induced up-regulation of DR5 and down-regulation of c-FLIP. However, other apoptosis related proteins (XIAP, survivin, DR4, Mcl-1, and Bcl-2) were not changed (Figure 1G). Taken together, these data suggest that garcinol enhances TRAIL-induced apoptosis in renal carcinoma Caki cells.

### 2.2. Garcinol Induces TRAIL Sensitization through Down-Regulation of c-FLIP Expression

**Figure 2:**
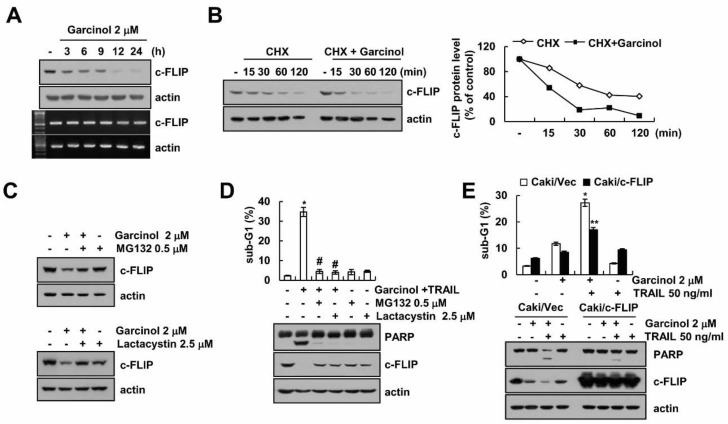
The effect of down-regulation of c-FLIP by garcinol treatment on TRAIL sensitization. (**A**) Caki cells were treated with 2 μM garcinol for 3–24 h. Western blot (upper) and RT-PCR (lower) showing the c-FLIP and actin; (**B**) Caki cells were treated with 20 μg/mL cycloheximide (CHX) in the presence or absence of 2 μM garcinol for 15–120 min. Western blot showing the c-FLIP and actin. The band intensity was calculated using Image J; (**C**) After pretreatment of 0.5 μM MG132 and 2.5 μM lactacystin for 30 min, Caki cells were treated with 2 μM garcinol for 24 h. Western blot showing the c-FLIP and actin; (**D**) After pretreatment of 0.5 μM MG132 and 2.5 μM lactacystin for 30 min, Caki cells were treated with 2 μM garcinol and 50 ng/mL TRAIL for 24 h. Levels of apoptosis were assessed by flow cytometry, and western blot showing the PARP, c-FLIP and actin; (**E**) Vector cells (Caki/Vec) and c-FLIP-overexpressed cells (Caki/c-FLIP) were treated with 2 μM garcinol and/or 50 ng/mL for 24 h. Levels of apoptosis were assessed by flow cytometry, and western blot showing the PARP, c-FLIP and actin. * *p* < 0.01 compared to the control. # *p* < 0.01 compared to the garcinol plus TRAIL. ** *p* < 0.01 compared to the garcinol plus TRAIL-treated Caki/Vec.

To investigate the role of c-FLIP protein in garcinol plus TRAIL-induced apoptosis, we examined mRNA and protein levels of c-FLIP in garcinol-treated cells. The mRNA levels of c-FLIP were not changed, but protein levels decreased in a time-dependent manner (Figure 2A). Next, we examined c-FLIP protein stability by garcinol treatment. The c-FLIP protein levels rapidly decreased in presence of cycloheximide (CHX), and were significantly lower in garcinol-treated cells than in vehicle-treated cells (Figure 2B). Pretreatment with proteasome inhibitors (MG132 and lactacystin) rescued the garcinol-mediated decrease of c-FLIP protein levels (Figure 2C), and inhibited induction of sub-G1 population and cleavage of PARP by combined treatment (Figure 2D). Therefore, our data suggested that garcinol decreases c-FLIP expression at post-translational levels in a proteasome-dependent pathway. Furthermore, ectopic expression of c-FLIP significantly inhibited apoptosis in garcinol plus TRAIL-treated cells (Figure 2E).

### 2.3. Combined Treatment Garcinol and TRAIL Induces Up-Regulation of DR5 Expression

**Figure 3:**
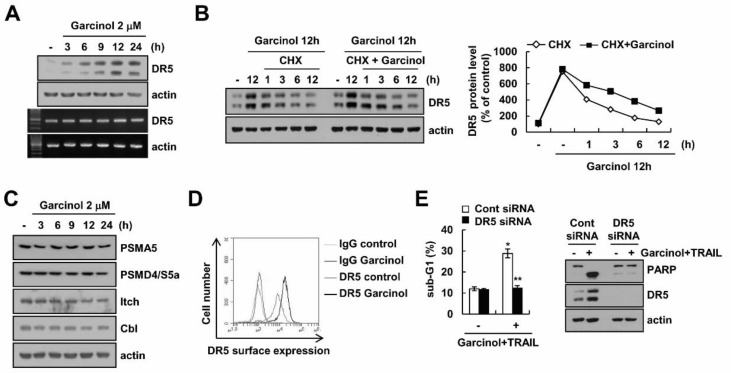
The effect of up-regulation of DR5 by garcinol on TRAIL sensitization. (**A**) Caki cells were treated with 2 μM of garcinol for 3–24 h. Western blot (upper) and RT-PCR (lower) showing the DR5 and actin; (**B**) After garcinol treatment for 12 h, Caki cells were treated with 20 μg/mL cycloheximide (CHX) in the presence or absence of 2 μM garcinol for 1–12 h. Western blot showing the DR5 and actin. The band intensity was calculated using Image J; (**C**) Caki cells were treated with 2 μM garcinol for 3–24 h. Western blot showing the PSMA5, PSMD4/S5a, Itch, Cbl and actin; (**D**) For DR5 surface staining, cells were treated with 2 μM garcinol for 24 h and the cell surface expression level of DR5 was determined by flow cytometry as mentioned in a material and methods; (**E**) Caki cells were transfected with control or DR5 siRNA, and then cells were treated with 2 μM garcinol plus 50 ng/mL TRAIL for 24 h. Levels of apoptosis were assessed by flow cytometry, and western blot showing the PARP, DR5 and actin. * *p* < 0.01 compared to the control. ** *p* < 0.01 compared to the garcinol plus TRAIL-treated control siRNA.

Garcinol induced up-regulation of DR5 protein levels, but mRNA levels of DR5 were not alterd (Figure 3A). Garcinol markedly enhanced DR5 protein stability (Figure 3B). Next, we investigated whether up-regulation of proteasome subunits was involved in garcinol-induced DR5 up-regulation. The expression levels of two critical proteasome subunits (PSMA5 and PSMD4/S5a) were not changed by garcinol treatment (Figure 3C). In addition, expression of E3 ligases (Itch and Cbl) for DR5 also did not change in garcinol-treated cells (Figure 3C). Therefore, we need further study to identify the E3 ubiquitin ligase or deubiquitinase involved in garcinol-induced DR5 protein up-regulation. Garcinol also increased surface expression levels of DR5 (Figure 3D). To investigate the role of DR5 up-regulation in garcinol plus TRAIL-induced apoptosis, cells were transfected with DR5 siRNA. Knock-down of DR5 expression by siRNA significantly inhibited apoptosis (Figure 3E).

### 2.4. Garcinol-Mediated TRAIL Sensitization Is Not Associated with Reactive Oxygen Species (ROS) Signaling Pathway

**Figure 4:**
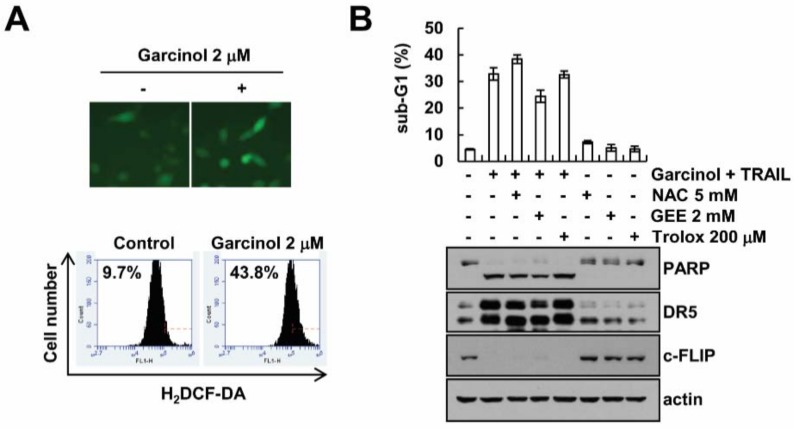
TRAIL sensitization by garcinol is independent of ROS levels. (**A**) For measurement of ROS production, cells were treated with 2 μM garcinol for 2 h and then stained with H_2_DCF-DA dye. Fluorescence was detected by fluorescence microscopy and flow cytometry; (**B**) After pretreatment with the indicated concentrations of NAC, GEE and trolox for 30 min, cells were stimulated with 2 μM garcinol plus 50 ng/mL TRAIL for 24 h. Levels of apoptosis were assessed by flow cytometry, and western blot showing the PARP, DR5, c-FLIP and actin.

Generation of ROS is linked to the TRAIL-mediated apoptosis [18,19]. Garcinol induced generation of ROS which was analyzed by H_2_DCF-DA-based fluorescence microscopy and FACS (Figure 4A). Therefore, we examined whether ROS are involved in TRAIL sensitization by garcinol treatment. ROS scavengers (NAC, GEE and Trolox) did not abolish garcinol plus TRAIL-induced apoptosis and cleavage of PARP (Figure 4B), and modulation of DR5 and c-FLIP expression (Figure 4B). Therefore, garcinol-induced TRAIL sensitization is independent of ROS signaling.

### 2.5. Garcinol Induces TRAIL Sensitization in Other Cancer Cells, but Garcinol Plus TRAIL Had No Effect on Apoptosis in Normal Cells

**Figure 5:**
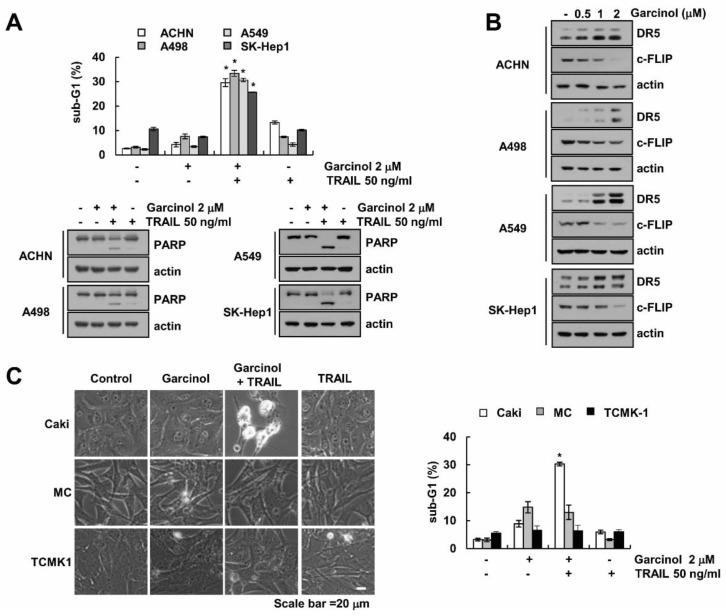
The effects of garcinol on TRAIL sensitization in cancer and normal cells. (**A**) ACHN, A498 (renal carcinoma), A549 (lung carcinoma) and SK-Hep1 (liver carcinoma) cells were treated with 2 μM garcinol and/or 50 ng/mL TRAIL for 24 h. Levels of apoptosis were assessed by flow cytometry, and western blot showing the PARP and actin; (**B**) Cells were treated with 0.5–2 μM garcinol for 24 h. The related levels of proteins were detected by western blot using indicated antibody; (**C**) Caki, masangial cells (MC) and TCMK-1 cells were treated with 2 μM garcinol and/or 50 ng/mL TRAIL for 24 h. Cell morphology was explored using an interference optical microscope. Levels of apoptosis were assessed by flow cytometry. * *p* < 0.01 compared to the control.

Next, we investigated whether garcinol also induces TRAIL sensitization in other types of cancer cells and normal cells. Garcinol induced TRAIL sensitization in other renal carcinoma (ACHN and A498), lung carcinoma (A549), and hepatoma (SK-Hep1) cells (Figure 5A). Expression patterns of DR5 and c-FLIP in other cancer cells are similar to that of Caki cells (Figure 5B). However, garcinol did not alter the sensitivity against TRAIL treatment in normal human mesangial cells (MC) and normal mouse renal tubular epithelial (TCMK-1) (Figure 5C). These data indicate that garcinol enhances TRAIL-mediated apoptotic cell death in cancer cells.

## 3. Discussion

Here, we found that garcinol enhanced TRAIL sensitization in renal carcinoma cells, and induced up-regulation of DR5 and down-regulation of c-FLIP. Combined treatment with garcinol plus TRAIL induced apoptotic cell death in cancer cells, but not in normal cells. These results suggest that garcinol could be an attractive TRAIL sensitizer.

Garcinol induced apoptosis in several cancer cell lines through multiple signaling pathways. The apoptotic effects of garcinol were correlated with inactivation of STAT-3, NF-κB, and PI3K/Akt signaling pathways [4,5,6]. However, whether garcinol exhibits similar activity in renal carcinoma cell lines has not yet been evaluated. Garcinol revealed cytotoxicity with IC_50_ of 10~20 μM in colorectal cancer cell line HT-29 and breast cancer cell line MCF-7, respectively [5,6]. In our study, low concentration of garcinol (2 μM) did not induce apoptotic cell death at 24 h in Caki cells. However, combined treatment garcinol (1–2 μM) and TRAIL caused apoptotic cell death in Caki, ACHN, A498, A549, and SK-Hep1 cells, but not normal cells. Prasad et al., also reported that garcinol potentiated TRAIL-induced apoptosis through modulation of death receptors and anti-apoptotic proteins [9]. Interestingly, they reported that the sensitizing effect of garcinol was mediated through ROS generation in HCT116 cells. However, ROS scavengers (NAC, GEE and trolox) did not inhibit combined treatment with garcinol plus TRAIL-induced apoptosis in our cell culture conditions (Figure 4B). We assumed that this discrepancy was caused by different cell types or experimental conditions. Prasad’s data showed that inhibitory effect of ROS scavengers only detected at high concentration of 10 mM NAC and 10 mM GSH [9]. High concentration of ROS scavengers induced unexpected results. In addition, we used a low concentration of garcinol (2 μM), which did not induce cell death. However, they used high concentration of garcinol (15 μM). High concentrations of garcinol generate more ROS than our systems. Thus, relative amounts of ROS result in different effects on cell death. Furthermore, multiple studies suggested that down-regulation of c-FLIP may play a critical role in TRAIL-induced apoptosis. Recently, our group reported that cathepsin S inhibitor induced down-regulation of c-FLIP expression through up-regulation of Cbl expression [20]. Cathepsin S inhibitor-induced mitochondrial ROS generation played a critical role in c-FLIP down-regulation [20]. ROS play important roles in post-translational regulation of c-FLIP protein through multiple mechanisms [18,19]. Therefore, we need further experiments to identify the ROS-dependent or -independent mechanisms involved in garcinol-induced modulation of apoptosis-related proteins.

In addition, we demonstrated that combined treatment with garcinol plus TRAIL induced up-regulation of DR5 and down-regulation of c-FLIP expression through modulation of protein stability. Garcinol did not affect Itch and Cbl E3 ligase expression. Recently, Oh et al., reported that knock-down of monocyte chemotactic protein-induced protein-1 (MCPIP1) suppresses DR5 deubiquitination [21]. However, we could not rule out the possibility of involvement of other E3 ligase and de-ubiquitinases. There are clearly different from the effects of garcinol on protein stability in DR5 and c-FLIP expression. Therefore, which E3 ligases or de-ubiquitinases are involved in the regulation of DR5, and c-FLIP requires further study. Taken together, our data showed that garcinol sensitized TRAIL-induced apoptosis in various cancer cells, but not in normal cells. Therefore, our data suggest that garcinol might be an attractive sensitizer for TRAIL resistance cancer cells.

## 4. Materials and Methods

### 4.1. Cell Cultures and Materials

Mouse kidney cells (TCMK-1) were a gift from Dr. T.J. Lee (Yeungnam University, Korea). Primary culture of human mesangial cells (Cryo NHMC) were purchased from Clonetics (San Diego, CA, USA), and other cell lines are from the ATCC (Manassas, VA, USA). All cells were maintained in Dulbecco’s modified Eagle’s medium, and supplemented with 10% FBS, 5% antibiotic solution, and 100 μg/mL gentamycin. Garcinol and lactacystin was purchased from Enzo Life Sciences (Ann Arbor, MI, USA). Recombinant human TRAIL and z-VAD-fmk were purchased from R&D (Minneapolis, MN, USA). Calbiochem supplied *N*-acetyl-l-cysteine (NAC), trolox, and MG132 (San Diego, CA, USA). The following antibodies were used: anti-XIAP, -Bcl-2, -Mcl-1, -survivin, -Cbl, and -Itch (Santa Cruz Biotechnology, Santa Cruz, CA, USA); anti-c-FLIP (ALEXIS Corporation, San Diego, CA, USA); anti-PARP, -DR5, -PSMA5, -PSMD4/S5a, -cleaved caspase-3 (Cell Signaling Technology, Beverly, MA, USA); anti-caspase-3 (Enzo Life Sciences, Ann Arbor, MI, USA); anti-DR4 (Abcam, Cambridge, MA, USA); anti-actin (Sigma-Aldrich, St. Louis, MO, USA). Santa Cruz Biotechnology (Santa Cruz, CA, USA) and Bioneer (Daejeon, Korea) supplied DR5 siRNA and GFP siRNA (control siRNA), respectively. Sigma Chemical Co. supplied other reagents (St. Louis, MO, USA).

### 4.2. Flow Cytometry and Western Blot Analysis

We performed the western blot using the RIPA lysis buffer [22,23] and flow cytometry analysis as described in our study [20].

### 4.3. 4′,6′-Diamidino-2-Phenylindole Staining (DAPI) Staining and DNA Fragmentation Assay

For detection of DAPI staining and DNA fragmentation, we used 300 nM DAPI solution (Roche, Mannheim, Germany), and the cell death detection ELISA plus kit (Boehringer Mannheim, Indianapolis, IN, USA) [20].

### 4.4. Detection of Caspase-3 Activity

To evaluate caspase-3 activity, cell lysates were obtained in 100 μL of reaction buffer and then measured activities with a spectrophotometer at 405 nm [20].

### 4.5. Reverse Transcription-Polymerase Chain Reaction (RT-PCR)

To obtain cDNA, total RNA was prepared using the TriZol reagent (Life Technologies; Gaithersburg, MD, USA), and used M-MLV reverse transcriptase (Gibco-BRL; Gaithersburg, MD, USA). For PCR, we used DNA polymerase with primers targeting actin , c-FLIP, and DR5 [20].

### 4.6. Detection of DR5 on Cell Surface

Detached cells by 0.2% EDTA were washed with PBS, and then suspended in 100 μM PBS including 10% FCS and 1% sodium azide, and added to the primary antibody (DR5-phycoerythrin; Abcam, Cambridge, MA, USA) for 1 h at room temperature. Then, the cells washed with PBS including 10% FCS and 1% sodium azide, and suspended in 400 μL of PBS for the detection of DR5 on cell surface by flow cytometry.

### 4.7. Detection of Reactive Oxygen Species (ROS) Production

We detected levels of ROS production using 2′, 7′-dichlorodihydrofluorescein diacetate (H_2_DCF-DA). Cells were incubated with 10 μM H_2_DCF-DA for 10 min, and then fluorescence was detected.

### 4.8. Statistical Analysis

The values in all graphs represent the mean ± SD from three independent samples. The data were analyzed using a one-way ANOVA and post-hoc comparisons (Student-Newman-Keuls) using the SPSS 22.0 software (SPSS Inc., Chicago, IL, USA).

## Abbreviations

TRAIL	Tumor necrosis factor-related apoptosis-inducing ligand
STAT	Signal transducer and activator of transcription
NF-κB	Nuclear factor-κB
PI3K	Phosphatidylinositol-4,5-bisphosphate 3-kinase
ROS	Reactive oxygen species
DR	Death receptor
cFLIP	Cellular FADD-like IL-1β-converting enzyme-inhibitory protein
CHX	cycloheximide
PSMA5	20S proteasome subunit type 5
PSMD4/S5a	26S proteasome non-ATPase regulatory 4
